# Selection and evaluation of reference genes for improved interrogation of microbial transcriptomes: case study with the extremophile *Acidithiobacillus ferrooxidans*

**DOI:** 10.1186/1471-2199-10-63

**Published:** 2009-06-25

**Authors:** Pamela A Nieto, Paulo C Covarrubias, Eugenia Jedlicki, David S Holmes, Raquel Quatrini

**Affiliations:** 1 ICBM, Universidad de Chile, Santiago, Chile; 2 Center for Bioinformatics and Genome Biology, Fundación Ciencia para la Vida, MIFAB, Santiago, Chile; 3 Depto. de Ciencias Biologicas, Facultad de Ciencias de la Salud, Universidad Andres Bello, Santiago, Chile

## Abstract

**Background:**

Normalization is a prerequisite for accurate real time PCR (qPCR) expression analysis and for the validation of microarray profiling data in microbial systems. The choice and use of reference genes that are stably expressed across samples, experimental conditions and designs is a key consideration for the accurate interpretation of gene expression data.

**Results:**

Here, we evaluate a carefully selected set of reference genes derived from previous microarray-based transcriptional profiling experiments performed on *Acidithiobacillus ferrooxidans *and identify a set of genes with minimal variability under five different experimental conditions that are frequently used in Acidithiobacilli research. Suitability of these and other previously reported reference genes to monitor the expression of four selected target genes from *A. ferrooxidans *grown with different energy sources was investigated. Utilization of reference genes *map*, *rpoC*, *alaS *and *era *results in improved interpretation of gene expression profiles in *A. ferrooxidans*.

**Conclusion:**

This investigation provides a validated set of reference genes for studying *A. ferrooxidans *gene expression under typical biological conditions and an initial point of departure for exploring new experimental setups in this microorganism and eventually in other closely related Acidithiobacilli. The information could also be of value for future transcriptomic experiments in other bacterial systems.

## Background

Gene expression interrogation, both at single-gene (classical gene expression analysis) and genome-wide (global transcriptome analysis) levels, are prominent fields of study. By providing quantitative measures of mRNA changes, it has the extraordinary potential of identifying the functional consequences of genetic variability and environmental influence. Accurate quantification of gene expression is providing great insight into the physiology and metabolic complexity of microbes and their consortia and is thus contributing to our understanding of both fundamental and applied issues of major interest.

Microarray transcript profiling is the most widely used technique to evaluate global gene expression in microbial systems. However, due to methodological uncertainties inherent to the technique, it is imperative to validate the expression of key genes by an alternative procedure and quantitative real-time PCR (qPCR) has become the method of choice. qPCR is accurate, exhibits a broad dynamic range, and is sensitive and reproducible [1-6]. However, when performing qPCR, several parameters need to be controlled in order to obtain accurate and reliable expression measurements; these include variations in the amounts of starting material between samples, RNA extraction efficiency, RNA integrity/quality, efficiency of cDNA synthesis, and differences in the overall transcriptional activity of the cells analyzed [7].

The most frequently used strategy to control for such variations is relative normalization, where the expression of a target gene is measured with respect to total RNA, rRNA or a stably expressed internal reference gene. The use of rRNA or other reference gene has the advantage that their expression permits normalization against the cumulative errors of the entire process [8]. Ideally, the reference gene should be universally valid, expressed stably and at a similar level across all samples, cells, experimental treatments, and designs. Unfortunately, no such reference gene has been identified [9-12] and even widely used control genes have proven unsuitable in certain situations [6,13,14].

Housekeeping genes are usually chosen as reference genes both in eukaryotes [15] and prokaryotes [16] because they are assumed: a) to be essential, b) to be ubiquitous, c) not to be regulated or influenced by the experimental procedure and d) to be expressed at similar levels in different types of cells. However, it is becoming increasingly clear that some commonly chosen housekeeping genes vary considerably across cell types, with time, or due to experimental treatment [13]. This may be explained partially by the fact that some housekeeping proteins participate in other functions as well as in basal cell metabolism [17-19].

Therefore, for an accurate comparison of mRNA transcription in different samples, either validated reference genes are required for normalization or new ones should be determined empirically for each experimental system and condition studied [20,21]. Here, we evaluate a carefully selected set of reference genes for improving the interpretation of gene expression profiles in a model microorganism, *Acidithiobacillus ferrooxidans*. The implementation of high-throughput microarray analyses of *A. ferrooxidans *[22,23] and PCR techniques for expression profiling [24-37], have greatly enhanced our understanding of the genetic and physiological potential of this bioleaching bacterium. However, suitable reference genes to evaluate gene expression have not previously been identified in *A. ferrooxidans*.

In order to address this lacuna, we screened high-density oligonucleotide array-based expression profiles available for *A. ferrooxidans *and identified a set of nine genes with minimal variability under different experimental setups. Quantitative real-time PCR was then used to determine the mRNA levels of these genes, comparing their transcription in five different experimental conditions. Finally, we evaluated the suitability of these and other previously reported reference genes to monitor the expression of four selected target genes from *A. ferrooxidans *grown with different energy sources. This study defines reference genes for normalization of gene expression for future research, sparing other researchers in this and related fields from cumbersome and time-consuming screenings for an ideal reference gene, provided that they verify the stability of these candidates under their conditions of study.

## Results & Discussion

### Selection of candidate reference genes

**Figure 1:**
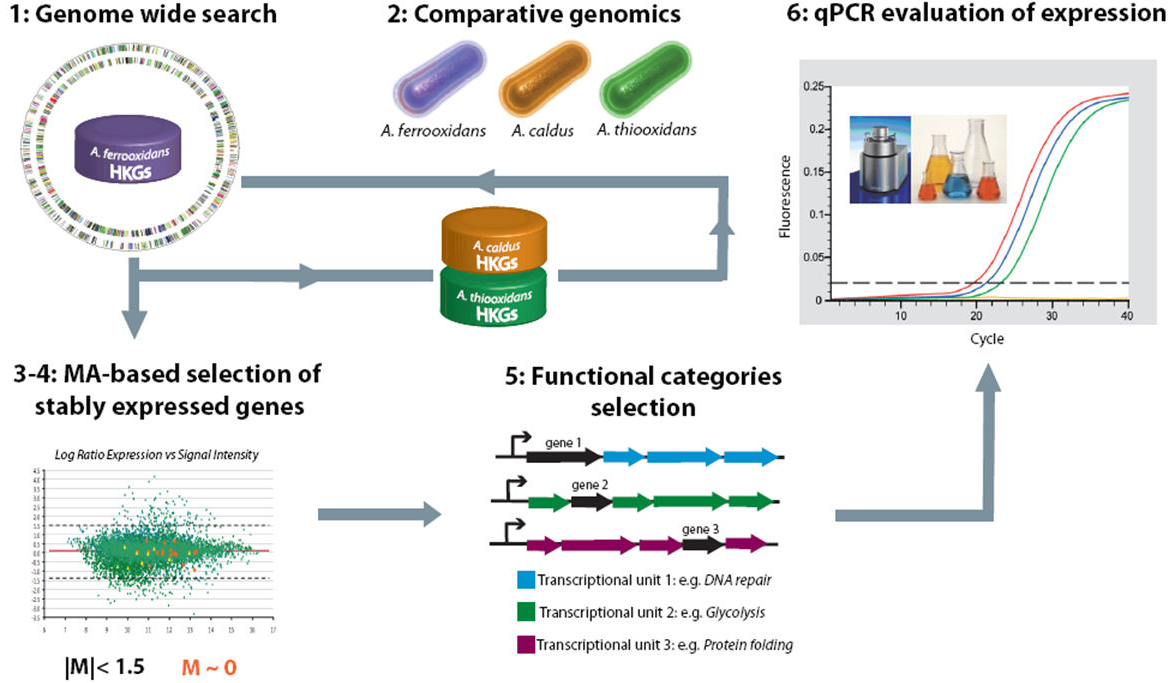
**Pipeline for the computational and experimental strategy used to identify suitable reference genes for normalization of expression**.

The most useful reference genes for standards in gene expression studies should be stably expressed over a range of experimental conditions and should be of wide phylogenetic distribution. Taking these requirements into account, a combined computational and experimental approach was devised to identify reference genes in the Acidithiobacilli (Figure 1). The strategy involves the following steps:

#### 1) A genome-wide bioinformatic identification of candidate reference genes

An initial set of candidate reference genes was compiled from a gene list that includes "housekeeping genes" of wide phylogenetic distribution [38]. This set was used to textmine the *A. ferrooxidans *ATCC 23270 genome (GenBank/EMBL/DDBJ accession number CP001219) [39]. Due to difference in ontological descriptions of gene function, not all genes could be recovered by textmining and additional candidates were identified in the *A. ferrooxidans *genome by BLASTP and TBLASTX searches using the housekeeping genes as queries. The combined set of candidate reference genes was then used to formulate bidirectional BLASTP and TBLASTX searches of the genomes of *A. thiooxidans *and *A. caldus*. This search for well conserved orthologs across Acidithiobacilli was performed in order to better define the set of essential genes for this bacterial genus. Such an approach will promote the ability to carry out future comparative gene expression studies within the Acidithiobacilli. Only genes present in all three genomes were accepted and provided the initial bioinformatic compilation of candidate reference genes (Additional File 1).

#### 2) The selection of stably expressed candidate reference genes for A. ferrooxidans

The expression profiles for the set of candidate reference genes was then evaluated in three different growth conditions of *A. ferrooxidans *(iron, pH 1.6 vs. sulfur, pH 3.5, iron-sulfur mixture, pH 1.6 vs. sulfur, pH 1.6 and high iron, pH 1.6 vs. low iron, pH 1.6; see Methods for more details). Candidate reference genes that exhibited non-differential expression (log ratio expression |M| < 1.5) and had the most similar level of expression (log ratio expression M~0) between every pair of conditions in all three experiments were further selected (Additional file 2).

#### 3) Removal of redundant candidate reference genes

The genetic context of these candidate reference genes in the genome of *A. ferrooxidans *was evaluated using the DNA sequence viewer and annotation tool Artemis v.10 [40]. In the case where more than one candidate gene belonged to the same operon or gene cluster, only one gene was selected for further experimental validation. Also, only one candidate was chosen from genes belonging to the same functional category as defined by TIGRfams [41]. This reduced redundancy in the set.

#### 4) Evaluation of the expression profiles of the candidate reference genes by real time PCR

The expression of these selected candidate reference genes was analyzed by quantitative PCR in order to evaluate if the stability of expression observed in microarray experiments was supported by more sensitive and rigorous evaluation methods. Transcriptional levels were compared by assessing C_t _values of each gene for two of the former experimental conditions (iron pH 1.6 and sulfur pH 3.5) and three new ones (sulfur pH 2.5, sulfur pH 4.5 and thiosulfate pH 4.5, see Methods for further detail). These conditions are frequently used in the laboratory to study the biology of Acidthiobacilli because they simulate environmental conditions. Expression values (C_t_) of the selected reference genes and their dispersion are plotted in Additional file 3.

The combined bioinformatics and experimental strategy identified nine candidate reference genes (*coaE, era, gmk, gyrA, map, nth, rplI, rpoC, trpS*).

### Expression stability of candidate reference genes

The expression of the 9 selected reference genes was analyzed in the five different experimental conditions described above by the methods of Vandesompele and Andersen as implemented in the Visual Basic Application geNorm [11] and Visual Basic Application NormFinder [7], respectively (Table 1). According to geNorm (which determines a gene expression stability value M to produce a rank where the best genes are those with the lowest M value) *map*, *rpoC*, *era *and *gmk *showed the least variability in expression in all conditions evaluated (range of expression of 1.029, 1.040, 1.046 and 1.067 fold respectively). The NormFinder approach (which enables identification of the single best genes in a ranking) showed *era *to be the most stable gene and *rpoC *and *gmk *to rank within the four most stably expressed genes. Slight differences observed between the two techniques are to be expected and can be explained by the way in which both methods analyze the data. geNorm selects the gene with the most stable expression independent of the expression of the other genes under analysis. NormFinder instead focuses on the genes with least intra- and inter-group expression variation, thus the selection of the best reference gene is affected by the other genes being analyzed. Similar differences between the results of geNorm and NormFinder have been reported in other studies [42,43]. Taking into account the results from both geNorm and NormFinder, we can conclude that *rpoC *and *era *are the most suitable reference genes for studies with the Acidithiobacilli.

**Table 1:** Expression stability ranking of candidate reference genes.

**Gene**	**geNorm**	**NormFinder**
***map***	0.258 (1)*	0.311 (6)
***rpoC***	0.258 (1)*	0.290 (3)
***era***	0.416 (2)	0.201 (1)*
***gmk***	0.477 (3)	0.299 (4)
***trpS***	0.542 (4)	0.306 (5)
***nth***	0.581 (5)	0.265 (2)
***rplI***	0.616 (6)	0.425 (8)
***gyrA***	0.664 (7)	0.412 (7)
***coaE***	0.763 (8)	0.628 (9)

Ranking of the nine evaluated reference genes according to their expression stability under five different experimental conditions (♣) using geNorm [11] and NormFinder [7] applications.

* Genes with the most stable expression values.

() The rank of the genes is indicated in parentheses.

(♣) Experimental conditions. Condition 1: cells grown in 9 K medium at pH 1.6 containing 200 mM FeSO_4_; condition 2: cells grown in 9 K medium at pH 2.5 containing 1% elemental sulfur; condition 3: cells grown in 9 K medium at pH 3.5 containing 1% elemental sulfur; condition 4: cells grown in 9 K medium at pH 4.5 containing 1% elemental sulfur; condition 5: cells grown in DSMZ71 medium at pH 4.5 containing 0.5% thiosulfate.

### Expression stability of reference genes previously used in studies of *A. ferrooxidans*

Three genes,* recA*, *alaS *and *rrs *(16S rRNA) have been used in prior studies as internal controls for experimental investigations in *A. ferrooxidans *[28,30,44,45], but there has been no formal report showing that they are reliable references. Conversely, there is evidence showing differential expression of *recA *and *rrs *under cellular stress [46] and starvation [45,47]. In addition, use of *rrs *as a reference gene has been challenged because it is a very abundant species of RNA present at concentrations outside most calibration ranges [16].

**Figure 2:**
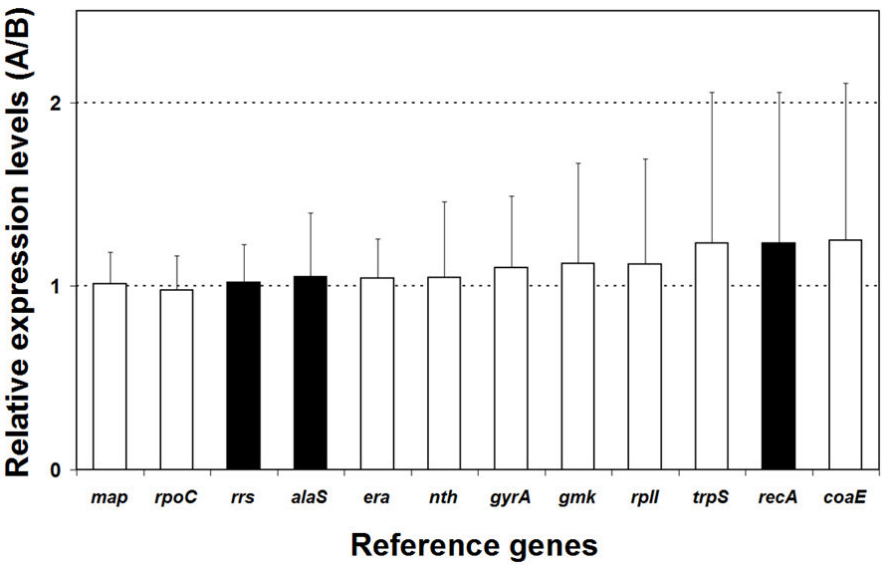
**Relative expression levels of *alaS*, *recA *and *rrs *and the nine candidate reference genes in all possible pairwise combinations (A/B) of five experimental conditions analyzed**. Expression levels of the reference genes in each experimental condition (A) was compared to their respective expression levels in all other conditions evaluated (B). Boxes represent the average ratios (A/B) in all possible pairwise combinations of experimental conditions analyzed and bars represent the standard deviations of the average ratios A/B. White boxes correspond to reference genes selected after applying the strategy outlined in Figure 1. Black boxes correspond to reference genes previously used in *A. ferrooxidans *research [28,30,45].

The expression of *recA*, *alaS *and *rrs *was analyzed under the same experimental conditions and their expression values were ranked with respect to the new reference genes derived above using geNorm [11] and NormFinder [7] (Table 2). The variability in expression of *rrs *and *alaS *in five different experimental setups shows them to perform well although slightly poorer than *rpoC*, *era *and *map *(Figure 2). Both geNorm and NormFinder identified *rrs *and *alaS *among the six more stable genes. On the contrary, *recA *ranks further down, indicating less stable expression, independently of the ranking method used (Table 2). In addition, the expression of *recA *varies more than two fold, and together with *coaE *and *trpS *exhibits the least suitable expression profile of the genes assessed in this study (Figure 2).

**Table 2:** Expression stability ranking of previously reported control genes.

**Gene**	**geNorm**	**Normfinder**
***rpoC***	0.258 (1)	0.310 (6)
***map***	0.258 (1)	0.331 (8)
***alaS***	0.299 (2)*	0.301 (5)*
***era***	0.411 (3)	0.229 (1)
***gmk***	0.487 (4)	0.295 (4)
***rrs***	0.522 (5)*	0.278 (3)*
***nth***	0.559 (6)	0.254 (2)
***trpS***	0.585 (7)	0.312 (7)
***rplI***	0.614 (8)	0.426 (10)
***gyrA***	0.657 (9)	0.525 (9)
***recA***	0.720 (10)*	0.476 (11)*
***coaE***	0.786 (11)	0.641 (12)

Ranking of the three control genes previously used in *A. ferrooxidans *gene expression studies according to their expression stability with respect to the nine candidate reference genes under five different experimental condition using geNorm and NormFinder applications. See Table 1 legend for further detail on the ranking methods and experimental conditions.

* Control genes used in previous studies [28,30,45].

() The ranks of the genes are indicated in parentheses.

It can be concluded that, among the previously used reference genes in *A. ferrooxidans*, only *alaS *is suitable and can be used with confidence as a normalizer. Given the variability observed in the present study, use of *recA *as a normalizer is not recommended as it would introduce noise to the analysis and eventually produce misleading results. In addition, use of *rrs *as a reference gene is not recommended despite its stable expression in the five conditions analyzed because its abundance prejudices the analysis of lowly abundant transcripts.

### Use of multiple reference genes for improved normalization

**Figure 3:**
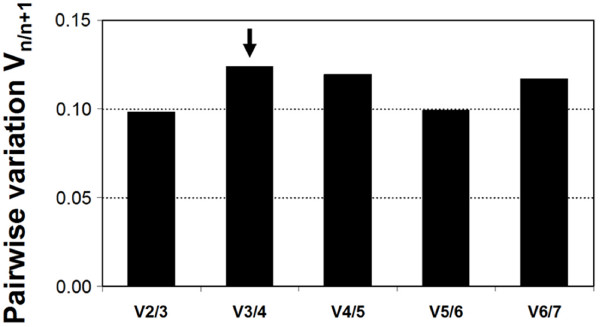
**Pairwise variation (V_n/n+1_) analysis between normalization factors (NF_n_/NF_n+1_) to determine the optimal number of reference genes required for normalization**.

Normalizing gene expression based upon the expression levels of a carefully selected set of reference genes, usually referred to as a normalization factor, performs better than normalizing against any single gene alone [11]. This raises the question as to whether a compromise could be discovered in which a pool of genes, fewer than nine but more than one, would provide greater confidence for use as a reference group in *A. ferrooxidans*. For this purpose, several normalization factors (NF) were calculated following the criteria defined by Vandesompele et al. [11]. A NF represents the geometric mean of *n *genes, and the pairwise variation between sequential normalization factors (NF_*n *_and NF_*n*+1_) gives an idea of how well each of these perform. Geometric means for seven of most stable genes, showing less than 1.5 fold variation in all experimental conditions (*map*, *rpoC*, *alaS*, *era*, *gyrA *and *nth*) were obtained and pairwise variations between any two subsequent values was calculated. As shown in Figure 3, addition of a fourth gene leads to a non-significant change in the average of the gene variance estimates. According to geNorm ranking, it is concluded that the NF derived from the pool of the three genes *map*, *rpoC *and *alaS *(NF_1_) is suitable for reliable normalization of gene expression of target genes. In spite of this we posit that *era*, which ranked first according to the Normfinder method, could also be included in the selected reference gene set.

### Use of selected reference genes to normalize expression of differentially expressed genes in Fe-S cells

To assess the value of our study, the relative expression levels of selected target genes was analyzed using the following normalization strategies: a) the three best reference genes selected by geNorm and Normfinder *rpoC*, *era *and *alaS *were used individually, b) a NF derived from the combination of the three genes selected by geNorm, *rpoC*, *map *and *alaS *(NF_1_), c) a NF derived from the combination of the top ranking genes selected by geNorm and Normfinder method *rpoC*, *map *and *era *(NF_2_) or d) the frequently cited reference genes *rrs *and *recA*. For this purpose, four target genes were selected that are known to be differentially expressed in *A. ferrooxidans *cultures: a) *sdrAI *(AFE0007) is 95 fold induced in iron [34], b) *cyoB *(AFE2407) is 12 fold induced in sulfur [22], c) *cbbOIa *(AFE1408) is 3 fold induced in iron [23] and d) *mntH *(AFE2920) is 24 fold induced in sulfur (unpublished results).

**Figure 4:**
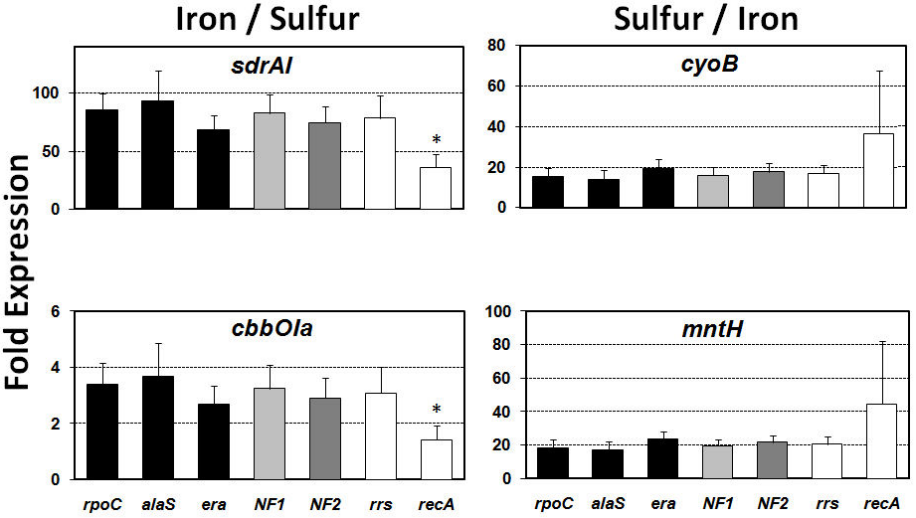
**Comparison of different normalization strategies**. Mean relative expression levels of four differentially expressed genes using different normalization strategies. Black: stable reference genes used individually, light grey: geometric mean of three stable reference genes selected by geNorm (NF_1_: *rpoC*, *map *and *alaS*), dark grey: geometric mean of four stable reference genes selected by geNorm (NF_2_: *rpoC*, *map *and *era*) and white: classical reference genes used individually. (*) Significantly different according to a two tailed unpaired t-test at 95% confidence.

Expression of the four genes of interest in iron versus sulfur grown cells was evaluated using the relative expression analysis software qBase v1.3.5 [48]. Figure 4 shows a significant increase in the expression of the *sdrA1 *and *cbbOIa *genes in cells grown in ferrous iron and of the *cyoB *and *mntH *genes in cells grown in sulfur. In all cases, normalization by individual reference genes outlined here (*rpoC*, *era *or *alaS*), by the geNorm derived NF_1 _(*rpoC *plus *map *plus *alaS*), by the combined NF_2 _(*rpoC *plus *map *plus *era*) and by *rrs *gave similar results. For example, *sdrA1 *was up-regulated 60–100 fold depending on whether the normalization strategy was a single stable reference gene or the normalization factors. Conversely, normalization by the *recA *gene dramatically altered the relative expression ratio of the target gene, revealing an up-regulation of less than 50.

These results demonstrate how the interpretation of bacterial gene expression levels can be affected by the choice of the reference genes in quantitative real-time RT-PCR analysis. If a single gene is to be used, e.g. in studies where only one or a few target genes are being evaluated, the reference gene should be one of the three validated stable reference genes *rpoC*, *era *or *alaS*. In investigations where a larger number of target genes are to be evaluated or a higher degree of confidence is desired, use of the NF derived from the pool of the genes *rpoC*, *map *and *alaS *or *era *is advisable.

## Conclusion

Normalization is a prerequisite for accurate real time PCR expression profiling. Significant random fluctuations or, even worse, directional changes in the expression of chosen reference genes between samples, can lead to the lack of detection of small differences between genes of interest or to erroneous results. Therefore, it is extremely important to find appropriate reference genes with minimal variability. This cumbersome task is often avoided and frequently priorly used reference genes are assumed to be good normalizers without further evaluation in unexplored experimental setups.

The geometric mean of few carefully selected genes, *rpoC*, *map, alaS *and/or *era*, is demonstrated to be the best normalizer for *A. ferrooxidans *in the diverse experimental conditions used in this study. Use of a single gene for normalization, instead, may result in relatively large variations in target gene expression and significant errors depending on the gene in question and the experimental setup, as showed to be the case when using the *recA *gene. Conversely, it is suggested that *rpoC*, *era *or *alaS *could be used as normalizers if only one reference gene is strictly necessary. Since ribosomal RNA is much more abundant than most target mRNA transcripts and its quantification falls outside most calibration ranges, the use of *rrs *is not recommended especially for the measurements of low abundance transcripts.

Whatever strategy is used to normalize for differences in quality and quantity of input RNA it must be validated for a particular experimental model on an individual basis. This investigation provides a validated set of reference genes for those studying *A. ferrooxidans *gene expression under typical biological conditions and an initial point of departure for those exploring new experimental setups in this microorganism or other closely related Acidithiobacilli or possibly also in other bacterial models.

## Methods

### Bioinformatic selection of candidate reference genes

An initial set of candidate reference genes was compiled from a gene list that includes "housekeeping genes" of wide phylogenetic distribution [38]. This set was used to textmine the *A. ferrooxidans *ATCC 23270 genome (GenBank/EMBL/DDBJ accession number CP001219) [39]. Additional candidates were identified in the *A. ferrooxidans *genome by BLASTP and TBLASTX searches. The combined set of candidate genes was then used to formulate bidirectional BLASTP and TBLASTX searches of the genomes of *A. thiooxidans *and *A. caldus*. Genomic context for genes present in all three genomes was analyzed using the DNA sequence viewer and annotation tool Artemis v.10 [40]. Candidate genes belonging to the same predicted operon or gene cluster were excluded from further analysis. Reference genes were classified by function using TIGRfams [41] and one gene per functional category was selected. These last two steps were included to reduce redundancy in the gene set.

### Bacterial strains and growth conditions

Gene expression was evaluated under the following experimental conditions: sulfur at pH 2.5, pH 3.5 and pH 4.5; thiosulfate at pH 4.5 and ferrous iron 200 mM pH 1.6. *A. ferrooxidans *strain ATCC 23270 was grown in modified 9 K basal salt media (0.7 mM (NH_4_)_2_SO_4_, 0.2 mM K_2_HPO_4_, 1.6 mM MgSO_4_.7H_2_O) containing iron (9 K + Fe: 200 mM FeSO_4_; adjusted to pH 1.6 with H_2_SO_4_) or sulfur (9 K + S: 1% ethanol-sterilized powdered sulfur, adjusted to pH 2.5; 3.5 and 4.5 with H_2_SO_4_). DSMZ71 medium was used for thiosulfate growth (20 mM Na_2_S_2_O_3_.5H_2_O, 22 mM KH_2_PO_4_, 2 mM MgSO_4_.7H_2_O, 22 mM (NH_4_)_2_SO_4 _and 1.7 mM CaCl_2_.2H_2_O). All cultures were incubated at 30°C under aerobic conditions on a rotary shaker at 150 r.p.m.

### Cell collection

*A. ferrooxidans *cultures to be used for nucleic acid purification were harvested at 8000 r.p.m. for 10 min. The cell pellet was washed in 9 K basal salt solution (adjusted at the corresponding pH). Washed cells were collected by centrifugation at 12000 r.p.m. for 10 min.

### DNA isolation

*A. ferrooxidans *cultures were grown for 72 h until stationary phase. DNA isolation was carried out by phenol-chloroform extraction. Briefly, cells were collected and resuspended in buffer TE (25:10) pH 8.0 with 5 mg/ml lysozyme, and incubated at 37°C for 30 minutes, followed by another hour of incubation at the same temperature with 1% SDS and 0.2 mg/ml proteinase K. Cell lysis was completed by alternate shifting of the suspension from 80°C to -80°C. DNA extraction was performed twice with a mixture of phenol:chloroform:isoamylic alcohol (25:24:1). Removal of the residual phenol was accomplished by one treatment with a mixture of chloroform:isoamylic alcohol (24:1). The DNA contained in the final aqueous phase was precipitated overnight at -20°C with absolute ethanol, washed with 70% ethanol, and finally resuspended in sterilized water. Genomic DNA quality and integrity were assessed by 1% (w/v) agarose gel electrophoresis and standard PCR, and concentration was determined by absorbance at 260 nm.

### Total RNA isolation

RNA was isolated from *A. ferrooxidans *mid-logarithmic cultures grown in modified 9 K basal salt medium in the presence of iron 200 mM, sulfur (1%, pH 2.5, 3.5 and 4.5) or 0.5% thiosulfate. Briefly, cells were collected and resuspended in ice-cold buffer TE (25:10) pH 8.0 with 1× Extraction Buffer (per liter: 1% SDS, 50 mM Tris-HCl pH 8.0, and 2 mM EDTA). Cell lysis was accomplished by incubation at 100°C for 5 minutes. The suspension was treated with TRIzol (Invitrogen), and the recovered aqueous phase was treated with chloroform followed by two extractions with acid phenol and chloroform. RNA was precipitated with absolute ethanol overnight at -20°C, washed with 70% ethanol, and finally resuspended in sterilized water. Samples were treated with DNase and purified with the Roche High Pure RNA Isolation Kit, following the manufacturer's recommendations and checked for DNA contamination by standard PCR, including a genomic DNA positive control. RNA quality was evaluated by 1.0% agarose gel electrophoresis and its concentration was measured by absorbance at 260 nm.

### Reverse transcription

cDNA was prepared from 1 μg total RNA using random hexamers and Superscript II reverse transcriptase (Invitrogen) according to manufacturer instructions. The resulting cDNA was diluted 1:10 in distilled water and stored in aliquots at -20°C until further use.

### Real-time PCR

Primers for real-time PCR assays and amplification efficiencies (E) are shown in Table 3. The real-time PCR reactions were performed in the Mx3000P QPCR System (Stratagene) using the SYBR GreenER qPCR SuperMix Universal Kit (Invitrogen). The 20 μl PCR reactions contained 2 μl of a 1:100 diluted cDNA sample; 200 nM of each primer and 1× SYBR GreenER qPCR SuperMix Universal (Invitrogen). The reference dye ROX was included at a final concentration of 5 nM. The cycling protocol was as follows: initial denaturation for 10 min at 95°C followed by 40 cycles of 30 s at 95°C, 15 s at 52°C; 30 s at 72°C. Fluorescence was measured after the extension phase at 72°C. The PCR products were subjected to a melting curve analysis, that commenced at 52°C and increased at 0.5°C s-1 up to 95°C, with a continuous fluorescent measurement. Specific amplification was confirmed by a single peak in the melting curve. For each experimental condition total RNA was extracted from two independent *A. ferrooxidans *cultures. Each RNA sample was retro-transcribed and the expression of all genes was assessed on the same cDNA sample. The reactions for each target gene were performed in triplicate and in the same PCR run. Thus, data sets consist of 6 values per gene per experimental set-up generated under standardized PCR cycling conditions. Stationary phase genomic DNA 10-fold dilutions (ranging from 10 ng to 1 pg) were used to generate a 5-point standard curve for every gene by using the Cycle Threshold (C_t_) value versus the logarithm of each dilution factor. Reaction efficiency (E = [10(-1/slope)]-1) for every gene was derived from the slope of the corresponding standard curves. Transcript quantities were calculated from the standard curve by the software accompanying the MxPro3000P QPCR System (Stratagene) set with default parameters. Each experiment included a no template control.

**Table 3:** Reference genes, qPCR primers and reaction parameters.

**Gene**	**Forward primer** **(5'-3')**	**Reverse primer** **(5'-3')**	**Amplicon** **size**	**Q-PCR Linear correlation** **coefficient**	**Q-PCR** **Efficiency (%)**	**Average** **Ct ± SD**
*Candidate reference genes*						

***coaE***	ACT ATC GCC CAT TGC TGG AT	TTG GTA ATG ATC CAG TCG GC	169	0.999	93	25.50 ± 0.69

***era***	CAT GGA TGA GAT CAA GAG CG	GTA TCC CGA GAA TCT GAT CC	174	0.999	94	22.56 ± 0.21

***gmk***	ATG GCA CTA GTG AAC CTT GG	AAT GAC CGT CTC CGA ATC CT	197	0.998	92	24.49 ± 0.50

***gyrA***	TAC CTC GAT TAC GCC ATG AG	TGT CAT AGA CAG CGG TAT CG	202	0.998	95	26.24 ± 0.45

***map***	TTA CCA CCG ATG AAC TGG AC	AGC CAT CCT TGA TAA CCG TG	222	0.999	94	20.64 ± 0.29

***nth***	ATC GTC TGG GAC TGT TCA AC	TAT TGA GGA CGA CAT TGG CG	153	0.997	94	25.31 ± 0.40

***rplI***	GAT GCC ATG GTT ACG ATT GC	GAC CAT CAC ATC CAG TTC GA	210	1.000	92	21.68 ± 0.51

***rpoC***	AAT GCG GTG TTG AGG TAA CC	AGG TAC TGG TCT TCG GTA AG	238	0.997	95	20.79 ± 0.23

***trpS***	TCT GCT CAT CGA ATG GTT GG	GAA TGT CTG CCG TCA TCA AC	230	0.999	89	23.86 ± 0.70

*Reference genes used in prior studies*						

***alaS***	CTG GAA TCT GGT CTT CAT GC	GCT TGA AGA GAT CGG TGT CA	152	1.000	94	22.35 ± 0.39

***recA***	CCG CCA ACA TTT CCC GGA CC	ACG CCG CGG TCC ACC AGT TC	301	0.998	98	29.41 ± 0.88

***rrs***	ACA CTG GGA CTG AGA CAC GG	ACC GCC TAC GCA CCC TTT AC	277	0.999	95	8.93 ± 0.27

*Differentially expressed target genes*						

***cbbOIa***	ACG GCA TTG AGC TAT ACC G	TCC GGG TCT AGT AGT GCA T	265	0.996	97	21.55 ± 0.72

***cyoB***	CAA TTA CAT GGT CCA CAA CA	AGC GTA TAC ACC ACG ATA CC	300	0.995	92	28.11 ± 2.17

***mntH***	ATA TCG GTG CCG TCA TCA TG	GAG AGC TGA TGC ACC GTA TT	217	0.996	97	21.92 ± 2.02

***sdrAI***	GTT TGG GTG CTG AAG TAG TG	GCA ACA GTG GCA AAC AGG C	255	0.995	93	23.61 ± 2.82

Primer sequences and reaction parameters used for quantitative real time PCR expression analysis.

### Stability of gene expression and relative quantification

The stability of gene expression was evaluated using the Excel-based applications geNorm [11] and Normfinder [7] and the relative expression was calculated with qBase 1.3.5 [48]. Briefly, the geNorm method is based on a pairwise comparison approach and depends on the calculation of an M value, defined as the average pairwise variation of a particular gene with all others. The NormFinder method is based on a different mathematic model that considers the intra- and inter-treatment variation in expression for gene ranking. The normalization factors were calculated following the criteria defined by Vandesompele [11]. These include: a) to use the geometric mean (n numbers are multiplied and then the nth root of the resulting product is taken) as this controls better for possible outliers and abundance differences between genes and b) to define the minimum number of reference genes needed for a reliable calculation by evaluating the pairwise variation between sequential normalization factors including three, four, five or more stable reference genes (NF_n_/NF_n+1_).

### Evaluation of reference gene expression by microarray transcript profiling

*A. ferrooxidans *gene expression was evaluated under three experimental conditions: (1) cells were grown in 9 K medium containing 62 mM FeSO_4 _at pH 1.6 versus cells grown in 9 K medium 1% elemental sulfur at pH 3.5 containing; (2) cells were grown in 9 K medium containing 62 mM FeSO_4 _plus 1% elemental sulfur at pH 1.6 versus cells grown in 9 K medium containing 1% elemental sulfur at pH 1.6 and (3) cells were grown in 9 K medium containing 200 mM FeSO_4 _at pH 1.6 versus cells grown in 9 K medium containing 62 mM FeSO_4 _at pH 1.6. Construction, experimental and data analysis protocols for *A. ferrooxidans *type strain specific oligonucleotide microarrays have been previously described [22] and deposited in the ArrayExpress database under the following accession numbers (A-MEXP-1478, A-MEXP-1479).

## Authors' contributions

PAN carried out the real time PCR standardization and analysis; PCC was responsible for the culture handling and preparation of the genomic DNA and total RNA samples. PAN and RQ conceived the study; EJ tutored PAN; RQ and DSH helped in the biological interpretation, and drafted the manuscript. All authors read and approved the final manuscript.

## Supplementary Material

Additional file 1 **Initial compilation of candidate reference genes**. Selection of conserved candidate reference genes belonging to different functional categories derived from textmining and blast searches in the genomes of three related Acidithiobacilli, *A. ferrooxidans *ATCC 23270, *A. thiooxidans *ATCC 19377 and *A. caldus *ATCC 51756. Click here for file

Additional file 2 **Candidate reference genes that survive culling after microarray transcript profiling**. Selection of conserved, non-differentially (|M| < 1.5) and stably (M~0) expressed reference genes belonging to different functional categories and different transcriptional units derived from a microarray dataset built upon 3 different experimental conditions. Condition A: cells grown in 9 K medium at pH 1.6 containing 62 mM FeSO_4 _(treatment) versus cells grown in 9 K medium at pH 3.5 containing 1% elemental sulfur (control); Condition B: cells grown in 9 K medium at pH 1.6 containing 62 mM FeSO_4 _plus 1% elemental sulfur (treatment) versus cells grown in 9 K medium at pH 3.5 containing 1% elemental sulfur (control); Condition C: cells grown in 9 K medium at pH 1.6 containing 200 mM FeSO_4 _(treatment) versus cells grown in 9 K medium at pH 1.6 containing 62 mM FeSO4 (control). Candidate reference genes are indicated in blue. Click here for file

Additional file 3 **Boxplot graph for the expression levels of candidate reference genes**. Comparison of the transcriptional expression levels of the nine candidate reference genes by direct plotting of the C_t _values (number of cycles needed for the fluorescence signal to reach a specific threshold level of detection). The C_t _median values for 5 different experimental setups are shown as lines, 25^th ^and 75^th ^percentile as boxes and ranges as bars. Condition 1: cells grown in 9 K medium at pH 1.6 containing 200 mM FeSO_4_, condition 2: cells grown in 9 K medium at pH 2.5 containing 1% elemental sulfur; condition 3: cells grown in 9 K medium at pH 3.5 containing 1% elemental sulfur; condition 4: cells grown in 9 K medium at pH 4.5 containing 1% elemental sulfur; condition 5: cells grown in DSMZ71 medium at pH 4.5 containing 0.5% thiosulfate. Candidate reference genes include: *gyrA*, DNA gyrase subunit A; *coaE*, dephospho-CoA kinase; *nth*, endonuclease III; *gmk*, guanylate kinase; *trpS*, tryptophanyl-tRNA synthetase; *era*, GTP-binding protein; *rplI*, ribosomal protein L9; *rpoC*, DNA-directed RNA polymerase subunit β and *map*, type I methionine aminopeptidase. Click here for file

## References

[B1] Klein D (2002). Quantification using real-time PCR technology: applications and limitations. Trends Mol Med.

[B2] Bustin SA (2000). Absolute quantification of mRNA using real-time reverse transcription polymerase chain reaction assays. J Mol Endocrinol.

[B3] Bustin SA (2002). Quantification of mRNA using real-time reverse transcription PCR (RT-PCR): trends and problems. J Mol Endocrinol.

[B4] Ginzinger DG (2002). Gene quantification using real-time quantitative PCR: an emerging technology hits the mainstream. Exp Hematol.

[B5] Heid CA, Stevens J, Livak KJ, Williams PM (1996). Real time quantitative PCR. Genome Res.

[B6] Gibson UE, Heid CA, Williams PM (1996). A novel method for real time quantitative RT-PCR. Genome Res.

[B7] Andersen CL, Jensen JL, Orntoft TF (2004). Normalization of real-time quantitative reverse transcription-PCR data: a model-based variance estimation approach to identify genes suited for normalization, applied to bladder and colon cancer data sets. Cancer Res.

[B8] Huggett J, Dheda K, Bustin S, Zumla A (2005). Real-time RT-PCR normalisation; strategies and considerations. Genes Immun.

[B9] Schmittgen TD, Zakrajsek BA (2000). Effect of experimental treatment on housekeeping gene expression: validation by real-time, quantitative RT-PCR. J Biochem Biophys Methods.

[B10] Vandecasteele SJ, Peetermans WE, Merckx R, Van Eldere J (2001). Quantification of expression of *Staphylococcus epidermidis *housekeeping genes with Taqman quantitative PCR during in vitro growth and under different conditions. J Bacteriol.

[B11] Vandesompele J, De Preter K, Pattyn F, Poppe B, Van RN, De Paepe A, Speleman F (2002). Accurate normalization of real-time quantitative RT-PCR data by geometric averaging of multiple internal control genes. Genome Biol.

[B12] Radonic A, Thulke S, Mackay IM, Landt O, Siegert W, Nitsche A (2004). Guideline to reference gene selection for quantitative real-time PCR. Biochem Biophys Res Commun.

[B13] Sturzenbaum SR, Kille P (2001). Control genes in quantitative molecular biological techniques: the variability of invariance. Comp Biochem Physiol B Biochem Mol Biol.

[B14] Takle GW, Toth IK, Brurberg MB (2007). Evaluation of reference genes for real-time RT-PCR expression studies in the plant pathogen *Pectobacterium atrosepticum*. BMC Plant Biol.

[B15] Zhong H, Simons JW (1999). Direct comparison of GAPDH, beta-actin, cyclophilin, and 28S rRNA as internal standards for quantifying RNA levels under hypoxia. Biochem Biophys Res Commun.

[B16] Theis T, Skurray RA, Brown MH (2007). Identification of suitable internal controls to study expression of a *Staphylococcus aureus *multidrug resistance system by quantitative real-time PCR. J Microbiol Methods.

[B17] Petersen BH, Rapaport R, Henry DP, Huseman C, Moore WV (1990). Effect of treatment with biosynthetic human growth hormone (GH) on peripheral blood lymphocyte populations and function in growth hormone-deficient children. J Clin Endocrinol Metab.

[B18] Singh R, Green MR (1993). Sequence-specific binding of transfer RNA by glyceraldehyde-3-phosphate dehydrogenase. Science.

[B19] Ishitani R, Sunaga K, Hirano A, Saunders P, Katsube N, Chuang DM (1996). Evidence that glyceraldehyde-3-phosphate dehydrogenase is involved in age-induced apoptosis in mature cerebellar neurons in culture. J Neurochem.

[B20] Czechowski T, Stitt M, Altmann T, Udvardi MK, Scheible WR (2005). Genome-wide identification and testing of superior reference genes for transcript normalization in Arabidopsis. Plant Physiol.

[B21] Remans T, Smeets K, Opdenakker K, Mathijsen D, Vangronsveld J, Cuypers A (2008). Normalisation of real-time RT-PCR gene expression measurements in *Arabidopsis thaliana *exposed to increased metal concentrations. Planta.

[B22] Quatrini R, Appia-Ayme C, Denis C, Ratouchniak J, Veloso F, Valdes J, Lefimil C, Silver S, Roberto F, Orellana O (2006). Insights into the iron and sulfur energetic metabolism of *Acidthiobacillus ferrooxidans *by microarray transcriptome profiling. Hydrometallurgy.

[B23] Appia-Ayme C, Quatrini R, Dennis Y, Denizot F, Silver S, Roberto F, Veloso F, Valdes J, Cardenas J, Esparza M (2006). Microarray and bioinformatic analyses suggest models for carbon metabolism in the autotroph *Acidithiobacillus ferrooxidans*. Hydrometallurgy.

[B24] Appia-Ayme C, Guiliani N, Ratouchniak J, Bonnefoy V (1999). Characterization of an operon encoding two c-type cytochromes, an aa(3)-type cytochrome oxidase, and rusticyanin in *Thiobacillus ferrooxidans *ATCC 33020. Appl Environ Microbiol.

[B25] Guiliani N, Jerez CA (2000). Molecular cloning, sequencing, and expression of *omp*-40, the gene coding for the major outer membrane protein from the acidophilic bacterium *Thiobacillus ferrooxidans*. Appl Environ Microbiol.

[B26] Butcher BG, Rawlings DE (2002). The divergent chromosomal ars operon of *Acidithiobacillus ferrooxidans *is regulated by an atypical ArsR protein. Microbiology.

[B27] Levican G, Bruscella P, Guacunano M, Inostroza C, Bonnefoy V, Holmes DS, Jedlicki E (2002). Characterization of the *pet *I and *res *operons of *Acidithiobacillus ferrooxidans*. J Bacteriol.

[B28] Yarzabal A, Appia-Ayme C, Ratouchniak J, Bonnefoy V (2004). Regulation of the expression of the *Acidithiobacillus ferrooxidans rus *operon encoding two cytochromes c, a cytochrome oxidase and rusticyanin. Microbiology.

[B29] Ramirez P, Guiliani N, Valenzuela L, Beard S, Jerez CA (2004). Differential protein expression during growth of *Acidithiobacillus ferrooxidans *on ferrous iron, sulfur compounds, or metal sulfides. Appl Environ Microbiol.

[B30] Rivas M, Seeger M, Holmes DS, Jedlicki E (2005). A Lux-like quorum sensing system in the extreme acidophile *Acidithiobacillus ferrooxidans*. Biol Res.

[B31] Quatrini R, Lefimil C, Holmes DS, Jedlicki E (2005). The ferric iron uptake regulator (Fur) from the extreme acidophile *Acidithiobacillus ferrooxidans*. Microbiology.

[B32] Barreto M, Jedlicki E, Holmes DS (2005). Identification of a gene cluster for the formation of extracellular polysaccharide precursors in the chemolithoautotroph *Acidithiobacillus ferrooxidans*. Appl Environ Microbiol.

[B33] Acosta M, Beard S, Ponce J, Vera M, Mobarec JC, Jerez CA (2005). Identification of putative sulfurtransferase genes in the extremophilic *Acidithiobacillus ferrooxidans *ATCC 23270 genome: structural and functional characterization of the proteins. OMICS.

[B34] Bruscella P, Appia-Ayme C, Levican G, Ratouchniak J, Jedlicki E, Holmes DS, Bonnefoy V (2007). Differential expression of two *bc*_1 _complexes in the strict acidophilic chemolithoautotrophic bacterium *Acidithiobacillus ferrooxidans *suggests a model for their respective roles in iron or sulfur oxidation. Microbiology.

[B35] Rivas M, Seeger M, Jedlicki E, Holmes DS (2007). Second acyl homoserine lactone production system in the extreme acidophile *Acidithiobacillus ferrooxidans*. Appl Environ Microbiol.

[B36] Levican G, Katz A, de Armas M, Nunez H, Orellana O (2007). Regulation of a glutamyl-tRNA synthetase by the heme status. Proc Natl Acad Sci USA.

[B37] Vera M, Pagliai F, Guiliani N, Jerez CA (2008). The chemolithoautotroph *Acidithiobacillus ferrooxidans *can survive under phosphate-limiting conditions by expressing a C-P lyase operon that allows it to grow on phosphonates. Appl Environ Microbiol.

[B38] Gil R, Silva FJ, Peretó J, Moya A (2004). Determination of the core of a minimal bacterial gene set. Microbiol Mol Biol Rev.

[B39] Valdes J, Pedroso I, Quatrini R, Dodson RJ, Tettelin H, Blake R, Eisen JA, Holmes DS (2008). *Acidithiobacillus ferrooxidans *metabolism: from genome sequence to industrial applications. BMC Genomics.

[B40] Rutherford K, Parkhill J, Crook J, Horsnell T, Rice P, Rajandream MA, Barrell B (2000). Artemis: sequence visualization and annotation. Bioinformatics.

[B41] Haft DH, Selengut JD, White O (2003). The TIGRFAMs database of protein families. Nucleic Acids Res.

[B42] Paolacci AR, Tanzarella OA, Porceddu E, Ciaffi M (2009). Identification and validation of reference genes for quantitative RT-PCR normalization in wheat. BMC Mol Biol.

[B43] Hibbeler S, Scharsack JP, Becker S (2008). Housekeeping genes for quantitative expression studies in the three-spined stickleback *Gasterosteus aculeatus*. BMC Mol Biol.

[B44] McGrew DA, Knight KL (2003). Molecular design and functional organization of the RecA protein. Crit Rev Biochem Mol Biol.

[B45] He Z, Zhong H, Hu Y, Xiao S, Liu J, Xu J, Li G (2005). Analysis of differential-expressed proteins of *Acidithiobacillus ferrooxidans *grown under phosphate starvation. J Biochem Mol Biol.

[B46] Stevenson DM, Weimer PJ (2005). Expression of 17 genes in *Clostridium thermocellum *ATCC 27405 during fermentation of cellulose or cellobiose in continuous culture. Appl Environ Microbiol.

[B47] Wagner R (2002). Regulation of ribosomal RNA synthesis in *E. coli *: effects of the global regulator guanosine tetraphosphate (ppGpp). J Mol Microbiol Biotechnol.

[B48] Hellemans J, Mortier G, De Paepe A, Speleman F, Vandesompele J (2007). qBase relative quantification framework and software for management and automated analysis of real-time quantitative PCR data. Genome Biol.

