# Supplementary figures and images for: Selection and evaluation of reference genes for improved interrogation of microbial transcriptomes: case study with the extremophile Acidithiobacillus ferrooxidans
Source: BMC Mol Biol. 2009 Jun 25;10:63. doi: 10.1186/1471-2199-10-63 (PMC2713239; doi:10.1186/1471-2199-10-63)

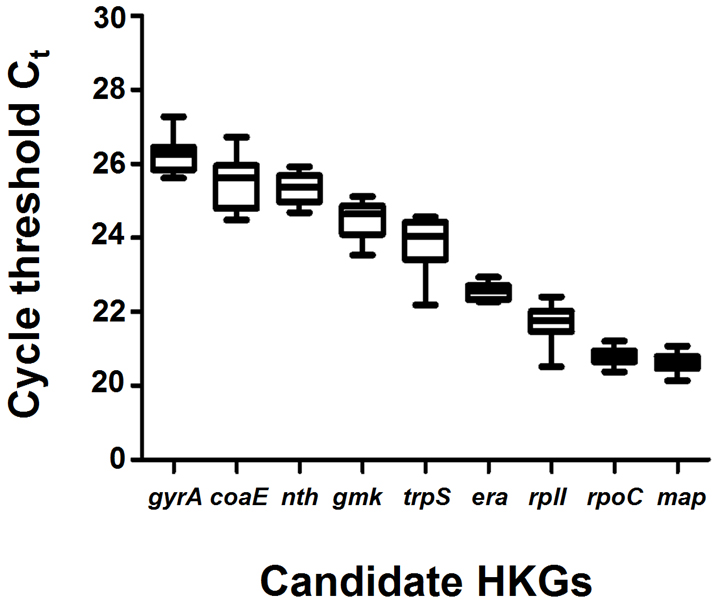

Supplement: Additional file 3 — Boxplot graph for the expression levels of candidate reference genes. Comparison of the transcriptional expression levels of the nine candidate reference genes by direct plotting of the Ct values (number of cycles needed for the fluorescence signal to reach a specific threshold level of detection). The Ct median values for 5 different experimental setups are shown as lines, 25th and 75th percentile as boxes and ranges as bars. Condition 1: cells grown in 9 K medium at pH 1.6 containing 200 mM FeSO4, condition 2: cells grown in 9 K medium at pH 2.5 containing 1% elemental sulfur; condition 3: cells grown in 9 K medium at pH 3.5 containing 1% elemental sulfur; condition 4: cells grown in 9 K medium at pH 4.5 containing 1% elemental sulfur; condition 5: cells grown in DSMZ71 medium at pH 4.5 containing 0.5% thiosulfate. Candidate reference genes include: gyrA, DNA gyrase subunit A; coaE, dephospho-CoA kinase; nth, endonuclease III; gmk, guanylate kinase; trpS, tryptophanyl-tRNA synthetase; era, GTP-binding protein; rplI, ribosomal protein L9; rpoC, DNA-directed RNA polymerase subunit β and map, type I methionine aminopeptidase. [file 1471-2199-10-63-S3.jpeg]
